# A Small Insulinomimetic Molecule Also Improves Insulin Sensitivity in Diabetic Mice

**DOI:** 10.1371/journal.pone.0169809

**Published:** 2017-01-10

**Authors:** Sandip Mukherjee, Mrittika Chattopadhyay, Sushmita Bhattacharya, Suman Dasgupta, Sahid Hussain, Saitanya K. Bharadwaj, Dhrubajyoti Talukdar, Abul Usmani, Bhola S Pradhan, Subeer S Majumdar, Pronobesh Chattopadhyay, Satinath Mukhopadhyay, Tushar K Maity, Mihir K. Chaudhuri, Samir Bhattacharya

**Affiliations:** 1 Cellular and Molecular Endocrinology Laboratory, Centre for Advanced Studies in Zoology, School of Life Science, Visva-Bharati (A Central University), Santiniketan, West Bengal, India; 2 Regional Centre for Biotechnology, NCR Delhi, India; 3 Department of Molecular Biology and Biotechnology, Tezpur University, Assam, India; 4 Department of Chemical Sciences, Tezpur University, Assam, India; 5 Division of Cellular Endocrinology, National Institute of Immunology, New Delhi, India; 6 Defence Research Laboratory, Tezpur, Assam, India; 7 Department of Endocrinology & Metabolism, Institute of Post-Graduate Medical Education & Research-Seth Sukhlal Karnani Memorial (IPGME&R−SSKM) Hospital, Kolkata, West Bengal, India; Brown University Warren Alpert Medical School, UNITED STATES

## Abstract

Dramatic increase of diabetes over the globe is in tandem with the increase in insulin requirement. This is because destruction and dysfunction of pancreatic β-cells are of common occurrence in both Type1 diabetes and Type2 diabetes, and insulin injection becomes a compulsion. Because of several problems associated with insulin injection, orally active insulin mimetic compounds would be ideal substitute. Here we report a small molecule, a peroxyvanadate compound i.e. DmpzH[VO(O_2_)_2_(dmpz)], henceforth referred as dmp, which specifically binds to insulin receptor with considerable affinity (KD-1.17μM) thus activating insulin receptor tyrosine kinase and its downstream signaling molecules resulting increased uptake of [^14^C] 2 Deoxy-glucose. Oral administration of dmp to streptozotocin treated BALB/c mice lowers blood glucose level and markedly stimulates glucose and fatty acid uptake by skeletal muscle and adipose tissue respectively. In *db/db* mice, it greatly improves insulin sensitivity through excess expression of PPARγ and its target genes i.e. adiponectin, CD36 and aP2. Study on the underlying mechanism demonstrated that excess expression of Wnt3a decreased PPARγ whereas dmp suppression of Wnt3a gene increased PPARγ expression which subsequently augmented adiponectin. Increased production of adiponectin in *db/db* mice due to dmp effected lowering of circulatory TG and FFA levels, activates AMPK in skeletal muscle and this stimulates mitochondrial biogenesis and bioenergetics. Decrease of lipid load along with increased mitochondrial activity greatly improves energy homeostasis which has been found to be correlated with the increased insulin sensitivity. The results obtained with dmp, therefore, strongly indicate that dmp could be a potential candidate for insulin replacement therapy.

## Introduction

Incidence of diabetes is threateningly increasing over the globe; it is strongly associated with cardiovascular disease, retinopathy and kidney failure. Impairment in insulin production or loss of insulin sensitivity in insulin target tissues causes diabetes mellitus. Type1 diabetes occurs when pancreatic β-cells are destroyed due to autoimmune disorder affecting considerable depletion in insulin secretion [1]. Hence, there is only one treatment option for Type1 diabetes i.e. insulin injection. Type2 diabetes on the other hand, is effected because of decreased tissue sensitivity to insulin. Oversupply of lipid is primarily responsible for producing this defect which leads to insulin resistance [2]. Interestingly, circulatory glycemic level or glucose homeostasis is not altered during insulin resistance because loss of insulin sensitivity is compensated by excess insulin secretion from pancreatic islet β-cells, when β-cells fail to meet the increasing demand, Type2 diabetes sets in [3,4].

Majority of presently available drugs for Type2 diabetes treatment target stimulation of insulin secretion from β-cells, for example sulfonylurea, sitagliptin, vidagliptin etc., while metformin reduces hepatic glucose production and increases glucose utilization. Most relevant drugs for Type2 diabetes are thiozolidinediones (TZDs) as they increase insulin sensitivity, however, their use has been restricted because of considerable adverse side effects [5]. At later stage of Type2 diabetes, β-cell dysfunction is very common [6], this consequently disrupts glycemic control due to dearth of insulin and only choice at this stage is insulin injection [7]. Demand for insulin therefore is substantially increasing for insulin replacement therapy. Insulin injection, may be more than one shot each day, becomes hazardous, inconvenient, causes tissue irritation, abscesses, allergy and discomfort [8].

In this report we demonstrate that a small molecule 3,5 dimethylpyrazole-peroxy-vanadate (dmp) binds to insulin receptor (IR) specifically and transduces insulin signal through the activation of IR that augments downstream insulin signaling thus effecting translocation of Glut4 to cell membrane which enhances glucose transport into the cell. Oral administration of dmp reaches blood within a short time, reduces blood glucose level in both Type1 diabetes and Type2 diabetes mice, restores energy homeostasis by stimulating mitochondrial biogenesis and improves insulin sensitivity through the augmentation of PPARγ and its target gene expression.

## Materials and Methods

### Reagents

Tissue culture materials were purchased from Gibco-BRL/Life Technologies, USA. 3T3L1 preadipocyte cell differentiation was done by using Adipogenesis Assay Kit, Cayman, Michigan, USA. [U-^14^C]-2- deoxyglucose (2-DOG) (Cat. No. NEC042V250UC; specific activity 250-360mCi/mmol), [9,10-^3^H(N)]-palmitate (Cat.No. NET043005MC; specific activity 30-60Ci/mmol) were purchased from GE Healthcare, Kowloon, Hong Kong. We procured primary antibodies for EGFR (sc-03), pEGFR (sc-23420-R), pIR-β (sc-81500), IR-β (sc-711), pIRS-1 (sc-17196), IRS-1 (sc-7200), pAkt (sc-7985), Akt (sc-8312), PPARϒ_2_ (sc-166731), Wnt3a (sc-136163), Adiponectin (Acrp 30, sc-17044-R), PGC-1 (sc-5816), NRF1 (sc-33771), mTFA (sc-23588) and α-Tubulin (sc-12462-R) from Santa Cruz Biotechnology Inc, CA, USA and AMPKα (#2532S), pAMPKα (#2535S) from Cell Signaling Technology, MA, USA. Alkaline phosphatase-conjugated goat anti-rabbit (A3687-1ML), rabbit anti-goat (A4187-1ML) and goat anti-mouse (A3562-1ML) were procured from Sigma Chemical Co, Louis, USA. Adiponectin ELISA Kit (ELM-Adiponectin,) was obtained from RayBiotech, Norcross, USA. Mice TNFα (Cat. No. ELM- TNFα) and IL-6 (Cat. No. ELM- IL-6) were purchased from RayBiotech, Norcross, USA. RT^2^-qPCR primers against mice GAPDH were procured from Qiagen, USA. Customized qPCR primers for PPAR-γ1 forward: 5'-AAGATTTGAAAGAAGCGGTGAAC-3' and reverse: 5'- CAATGGCCATGAGGGAGTTAG-3'; PPAR-γ2 forward: 5’-GCTGAATCACCCAGAGTCCTCTC-3’ and reverse: 5’- GGTGAAGGCTCATGTCTGTCTCTG-3’; Adpn forward: 5’ GCAAGTTCTACTGCAACATTCCGGG-3’ and reverse: 5’-GGTCGTAGGTGAAGAGAACGGCC-3’; CD36 forward: 5’-GTAGAGATGGCCTTACTTGGGATTGG-3’ and reverse: 5'- GCCAGTGTATATGTAGGCTCATCCAC-3'; Ap2 forward: 5’- GGGATTTGGTCACCATCCGGTCAG-3’ and reverse: 5’-CCCGCCATCTAGGGTTATGAT GC-3’; Wnt 3a forward: 5’-CCCAGAGGCACTGCTCTATC-3’ and reverse: 5’-TCCAAAAGTTCCACCCAGTC-3’; Axin2 forward: 5′-GAGTAGCGCCGTGTTAGTGACT-3′ and reverse: 5’-CCAGGAAAGTCCGGAAGAGGTATG-3′; Wisp2 forward: 5’-GTTTTGTGCCGCTGTGATG-3’ and reverse: 5’-CTGAGGAGGGCTGGATTG-3’; COXII forward: 5′-AATTGCTCTCCCCTCTCTACG -3′ and reverse: 5′-GTAGCTTCAGTATCATTGGTGC -3′ and RIP140 forward: 5’-CGGCCTCGAAGGCGTGG-3’ and reverse: 5’-AAACGCACGTCAGTATCGTC-3’ were purchased from IDT, USA.

### Animals and treatments

In this present study, we used control BL6 (C57BLKS/6J) and db/db (BKS.Cgm+/+Lepr(db)/J) female mice obtained from the Jackson Laboratory, aged 5–6 weeks, housed under a 12-h light/12-h dark cycle at 23 ± 2°C and relative humidity 55 ± 5% along with access to standard diet ad libitum. Female BALB/c mice 3-4months old of about 20–25 g weight were conditioned at 25 ± 2°C with a 12-h light/12-h dark cycle and fed on a standard diet (SD) ad libitum. A subset of control BALB/c mice were made insulin resistant by providing high fat diet (HFD) for 12weeks [9]. Another subset of control BALB/c mice were injected with a single dose of streptozotocin (STZ) intraperitoneally (50 mg kg^-1^ body weight dissolved in 50 mmol l^-1^ citrate buffer, pH 4.5) (Sigma-Aldrich, St. Louis, MO, USA) to induce diabetes. At the same time control BALB/c mice received an equal volume injection of citrate buffer. Weights and food intake were recorded every day. 72-hr after the injection of STZ or citrate buffer, blood glucose levels were assessed using a Accu-Chek glucometer (Roche, Basel, Switzerland). Only diabetic animals with glucose level > 300 mg dl^-1^ were used. dmp (300 μg kg bw^-1^) or vehicle (saline water) was orally administered to a subset of STZ mice for 6 h and db/db mice for 28 days. Triglyceride levels were estimated by Benasphera kit (Avantor performance materials, PA, USA), and free fatty acids by using Free Fatty Acid Quantitation Kit (Sigma,St. Louis, USA). GTT was assessed by estimating blood glucose concentration before and after oral gavages of 1g glucose kg bw^-1^at the indicated time points using Accu-Chek glucometer (Roche, Basel, Switzerland) and ITT was conducted in similar way by injecting 0.7U insulin kg bw^-1^. Insulin function test was evaluated by HOMA-IR and was calculated as fasting plasma glucose (mmol/l) x fasting serum insulin (mU/l) /22.5. All animal care and experimental procedures described in this study were specifically approved by the Animal Ethics Committees of Visva-Bharati (a Central University), Santiniketan (Permit No. 1819/GO/ERe/S/15/CPCSEA) and National Institute of Immunology, New Delhi (Permit No. IAEC#305/12). All surgery was performed under odium pentobarbital anesthesia, and all efforts were made to minimize suffering.

### Synthesis of the vanadium compound

To an aqueous solution of 0.5 g (2.76 mmol) of vanadium pentoxide, V_2_O_5_, in 5 ml of water, 2 mL (27 mmol) of 46% hydrogen peroxide was taken in a pre-cooled (*ca*. 0°C) 100 ml beaker. The reaction mixture being maintained at *ca*. 0°C was stirred till all the V_2_O_5_ dissolved and the solution became reddish-brown. To the clear solution dmpz was added maintaining the ratio V: dmpz as 1: 2.4. The pH of the solution at this stage was recorded to be *ca*. 5.5. It was stirred for 3 hours at ice cold condition and ethanol was added to initiate the precipitation. The product was obtained as bright yellow crystals. Well formed rod-shaped yellow crystal suitable for XRD analysis was obtained after two months from very dilute mother liquor. Yield of the product was 1.45 g (81%).

### Primary culture

Abdominal adipose tissue from control and treated mice were cultured according to the earlier protocol [9]. Briefly, abdominal adipose tissue was collected from control and treated mice and weighed and then rinsed with sterile 0.9% NaCl solution. After the adipose tissue was properly minced, it was digested with 3.3 mg ml^-1^type II collagenase in HBSS buffer containing 5.5 mM glucose, 5% fatty acid free BSA at 37°C in a shaking water bath for 30 min. Then it was filtered through two layers of nylon mesh followed by centrifugation at 300 g for 5 min. The floating cells were collected as adipocytes, washed twice and finally resuspended in serum free media (SFM). Soleus muscles from 2-3days old neonatal mice were cultured following our earlier method [10]. Briefly, soleus muscles were dissected out and incubated in 0.2% collagenase and 0.05% trypsin in PBS (phosphate buffered saline 0.05M, pH 7.5, 0.15M NaCl) with continuous stirring. The dispersed skeletal muscle cells were pelleted by centrifugation at 500g. They were then washed and resuspended in serum free media. The cells were plated in 6-well culture plates and dmp or insulin was added to the wells for different experiments. They were kept then in humidified 95%O_2_/5% CO_2_ atmosphere at 37°C for 4 h. For IR inhibition experiments, mice adipocytes were incubated with 100 μM of an intracellular IR tyrosine kinase inhibitor, HNMPA-(AM)3,(Cat# BML-EI248-0005, ENZO Life Sciences, NY, USA) for 1h prior to dmp or insulin addition according to earlier publication [11]. After that the cells and media were collected for further experiments.

### Cell cultures and treatments

L6 skeletal muscle cell line and mouse pre-adipocyte 3T3L1 were procured from the National Centre for Cell Science, Pune, India. Cells were cultured in DMEM containing penicillin (100U ml^-1^), streptomycin (100 μg ml^-1^), supplemented with 10% FBS and incubated at 37°C in humidified atmosphere with 5% CO_2_. Two days post confluence, 3T3L1 preadipocytes were differentiated over 5 days in differentiation medium supplemented with 5 μg ml^-1^ insulin, 0.5 mmol l^-1^ 3-isobutyl-1-methylxanthine and 1 μmol l^-1^dexamethasone. After differentiation, 3T3L1 adipocytes were washed thoroughly and used for different incubations in serum free media without antibiotics. To perform experiments using L6 myotubes, L6 myoblast were differentiated to L6 myotubes following earlier publications [12, 13]. Briefly, L6 myoblasts were cultured in DMEM supplemented with 10% FBS, blasticidin S (2μg ml^-1^), and 1% antibiotic/antimicotic solution (10,000 U ml^-1^ penicillin, 10 mg ml^-1^ streptomycin and 25μg ml^-1^ amphotericin B) under 5% CO_2_ at 37°C in a humidified chamber. After 2–3 passage of L6 myoblasts, cells were then allowed to grow and fuse into myotubes in the culture medium containing 2% FBS. Differentiation of myotubes was monitored under the microscope and all experiments were performed on maximally differentiated cells (>85%), at 7 days of post confluence. For in vitro experiments, 3T3L1 adipocytes and L6 myotubes were incubated with 50–300 nM of dmp or 20–120 nM insulin for 4 h. Viability of cells was assessed by MTT assay. To find out whether dmp regulate Wnt3a through IR, 3T3L1 adipocytes were preincubated for 1 h with 100 μM HNMPA-(AM)3, an intracellular insulin receptor tyrosine kinase inhibitor, followed by addition of 100 nM insulin or 250 nM dmp for 4h. IR autophosphorylation (PathScan^®^ Phospho-Insulin Receptor β (Tyr1150/1151) Sandwich ELISA Kit, Cell Signaling Technology, MA, USA) and tyrosine kinase activity (InsR Kinase Enzyme System, Promega, and ADP-GloTM Kinase Assay Kit, Promega, Wisconsin, USA) was performed according to manufacturer’s protocol. IR siRNA was transfected to L6 myotubes (2×10^5^ cell/well) by using Lipofectamine 2000 (Invitrogen, CA, USA) following manufacturer's protocol. After 48 h of transfection, cells were washed with DMEM and then used for different experiments.

### GLUT4 translocation assay

GFP-Glut 4 transfection was carried according to our earlier published method [10]. Briefly, L6 skeletal muscle cells were plated on 60 mm plate containing cover slips and maintained in an air/CO_2_ (19:1) atmosphere in DMEM containing 10% (v/v) FBS and 100μg ml^-1^ penicillin/streptomycin. After 8 h, cells were washed with DMEM free from FBS and then plasmid DNA of GFP-Glut 4 (2 μg) was used to transfect 2×10^5^ cells on each 60mm plate with Lipofectamine reagent in accordance with the manufacturer's protocol (Lipofectamine 3000 transfection kit, Invitrogen, CA, USA). After 48 h of transfection (transfection efficiency: 65–70%) cells were incubated without (control) or with insulin (100 nM) or dmp (250 nM) for 4 h. On termination of the incubation, cells on the cover slips were fixed in paraformaldehyde (3.5%) and mounted on glass slides. The cover slips were examined for translocation of GFP-Glut 4 under fluorescent microscope (Zeiss, Oberkochen, Germany).

### Surface Plasmon Resonance

The interaction of IR with dmp or Insulin (Cat No. 91077C, Sigma-Aldrich, St. Louis, USA) was determined by Surface Plasmon Resonance (SPR) analysis by Biacore T200 (GE Healthcare Life Sciences, MA, USA). Recombinant IR (Cat No. 1554-IR, R&D Systems, Inc. Minneapolis, USA) was immobilized in a CM5 sensor chip (Product Code: BR100012; GE Healthcare Life Sciences, MA, USA) by amine coupling method. Varying concentration of insulin or dmp was passed through the immobilized sensor chips with a flow rate of 30 μl min^-1^ over 120 seconds. The sensogram of IR-insulin or IR-dmp interactions demonstrated differential binding affinities. The equilibrium binding constant for IR and dmp was estimated using concentration dependent relative response data which was fitted with steady state affinity model.

### Fluorescence Quenching Measurement

Interaction of IR and vanadium compound, dmp was also determined by the fluorescence titration experiment. Room temperature tryptophan fluorescence was measured in recombinant IR with the concentration of 0.3 μM in 50 mM Phosphate buffer pH7.4, 50mM NaCl using Hitachi spectrofluorometer with the slit width of 10 nm. The emission spectra were recorded from 300 to 500 nm with an excitation wavelength 280 nm. Fluorescence intensity of vanadium compound was negligible in this specific excitation wavelength. The fluorescence intensities of IR under different concentration at 346 nm were monitored. The fluorescence quenching constant was estimated from Stern Volmer plot analysis.

### Immunoblotting

Adipocytes, skeletal muscle cells or cell pellets were resuspended in RIPA buffer containing protease inhibitor cocktail and 1mM PMSF, sonicated on ice and lysates were centrifuged for 10 min at 10,000g and protein concentrations of supernatant were determined following a previously described method [10]. Protein from tissue extract or cell lysates or media was resolved on 10% SDS-PAGE and then transferred to PVDF membranes (Millipore, Bedford, MA, USA) with the help of Wet/Tank Blotting System (Bio-Rad Laboratories Inc, Hercules, CA, USA). Membranes were probed with specific primary antibodies (1:1000) and then detected by using secondary antibody conjugated with alkaline phosphatise (1:3000). 5-bromro-4-chloro-3-indolyl phosphate/nitroblue tetrazolium (BCIP/NBT) was used for detection of the protein bands. Intensity of the bands was analyzed using Image Lab Software (Bio-Rad Gel DocTMXR+, USA). α-Tubulin was used as a loading control and it was not affected by incubation with insulin or dmp. Similarly, the amount of tubulin was not different among BL6, db/db and dmp treated db/db mice samples.

### [^14^C]2-DOG uptake

Briefly, cells were serum starved overnight in Kreb's Ringer Phosphate (KRP) buffer supplemented with 0.2% bovine serum albumin. Cells were treated with porcine-insulin (100 nM) or dmp (250 nM) for 25 min followed by addition of [^14^C] 2-DOG (0.4 nmol ml^-1^) for 5 min before the termination of incubation. Cells were harvested with trypsin–EDTA solution, solubilised with 1% NP-40. [^14^C] 2-DOG uptake was measured in a Liquid Scintillation Counter (Perkin Elmer, Tri-Carb 2800TR). For experiments with primary culture, skeletal muscle was treated with porcine-insulin (100 nM) or dmp (250 nM) for 25 min and then [^14^C] 2-DOG (0.4 nmol ml^-1^) was added for 5 min and [^14^C] 2-DOG uptake was measured for different time intervals.

### [^3^H]Palmitate uptake

Adipocytes were pretreated with insulin (100 nM) or dmp (250 nM) for 4h followed by incubation with 1μCi ml^-1^[^3^H] Palmitate for 15min. Cells were washed three times using ice-cold KRB buffer following solubilisation with 1% NP-40 and uptake of [^3^H] Palmitate was analyzed in a liquid scintillation counter (PerkinElmer Tri-Carb 2800TR, MA, USA) according to a previous description [14].

### Quantitative PCR

RNA was extracted from cells using RNeasy Lipid Tissue Mini Kit (Qiagen, Hilden, Germany) according to manufacturer’s instruction. RNA was then treated with DNase I and reverse transcribed with Revert Aid first strand cDNA synthesis kit (Fermentas, MA, USA). Gene-specific primers obtained from Qiagen and IDT were used to perform SYBR green-based real-time quantitative PCR (Applied Biosystems, CA, USA). Specificity of the products was determined by performing a melting curve analysis after the final extension. GAPDH was simultaneously amplified in separate reactions which were used for correction of Ct value. GAPDH was not affected by incubation with insulin or dmp.

### Isolation of mitochondria

Mitochondria were isolated from skeletal muscle tissue by differential centrifugation following an earlier published method with modifications [15, 16]. First the muscle tissue was cut into small pieces, digested with trypsin and then homogenised in a buffer containing 225 mM mannitol, 75 mM sucrose, 5 mM HEPES, 1 mM EGTA, and1 mg ml^-1^ BSA followed by centrifugation at 2000g for 5 min at 4°C. The supernatant was aspirated out and again centrifuged at 12,000 g for 10 min. The brown mitochondria pellet formed was then washed twice with 10 ml of washing buffer (225 mM mannitol, 75 mM sucrose, and 5 mM HEPES pH 7.4) at 12,000 g for 10 min at 4°C and then the final pellet was resuspended in isotonic buffer containing 145 mM potassium chloride, 50 mM sucrose, 1 mM EGTA, 1 mM magnesium chloride, and 10 mM phosphate buffer, pH 7.4 for measurement of ATP synthesis. Total DNA was extracted from muscle tissue and the content of mtDNA was calculated using real-time quantitative PCR by measuring the threshold cycle ratio (ΔCt) of a mitochondrial-encoded gene COXII versus a nuclear encoded gene RIP140.

### In vivo pharmacokinetic study

In-vivo pharmacokinetic release experiments of dmp was performed using C57BL/6 mice by orally administering dmp (5 mg kg bw^-1^) and then blood was collected at different indicated intervals of time. The concentration of dmp was estimated by following the method described by Willsky and his co-worker [17]. In order to evaluate C_max_, the time required to reach C_max_, (t_max_), elimination rate constant (K_el_) and concentration time curve (AUC) was estimated. The half-life (t_1/2_) was calculated by dividing 0.693 with K_el_ and by using the first order reaction kinetic C_t_ = C_o_ e−k (t–t_o_), where C_t_ is the serum concentration at time t, C_o_ is the initial concentration at time to, and k is the elimination constant.

### Determination of metabolic activities

Metabolic activities were measured by indirect calorimetry in BL6, db/db and dmp fed db/db mice. The metabolism rates were measured as oxygen consumption in closed-system in metabolic of 0.5 litre capacity. The experimental animals of three groups were placed in metabolic cage (INCO, India) and sealed accordingly. The average hourly oxygen consumption (V̇O_2_) was measured at ambient temperature of 25 ± 1°C as described earlier [18] and carbon dioxide production (V̇CO_2_) was measured according a previously published method [19]. V̇O_2_ and V̇CO_2_ were normalized with respect to body weight. The respiratory exchange ratio (RER) was calculated as the ratio of carbon dioxide production and oxygen consumption (V̇CO_2_/V̇O_2_). Energy expenditure (EE) was calculated on the basis of the formula [20], EE = 3.815 x V̇O_2_+ 1.232 x V̇CO_2_.

### Statistical analysis

One-way ANOVA was used to analyze the data where the *P* value indicated significance, means were compared by post hoc multiple range test. All values are means±s.e.m. We considered *p* value <0.05 as statistically significant.

## Results

### DmpzH[VO(O_2_)_2_(dmpz)]: the molecule

**Fig 1:**
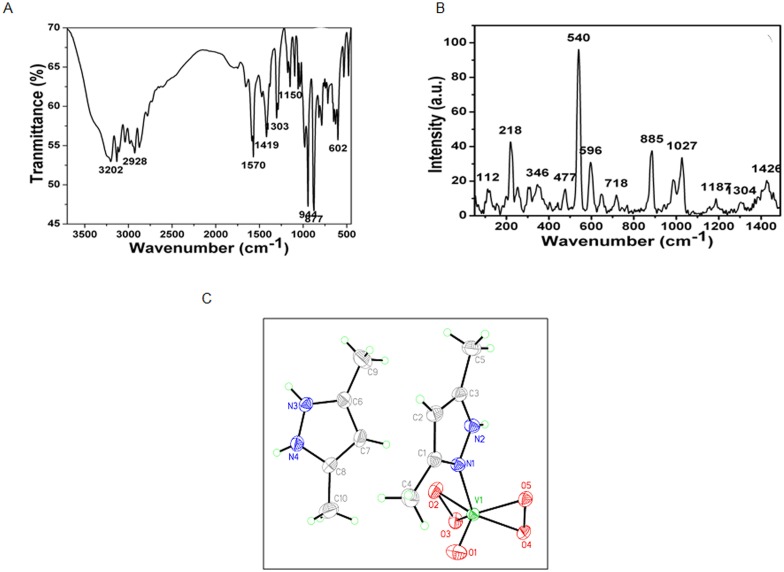
Structure of dmp. (A) FT-IR of DmpzH[VO(O2)2(dmpz)]. The symmetric O-O stretch, the symmetric metal-peroxo stretch, and the asymmetric metal-peroxo stretch, and occurred ca. 880, 600 and 500 cm^-1^, respectively. (B) Raman spectra of DmpzH[VO(O2)2(dmpz)]. The spectra shows strong bands at ~988.66, ~881.00, ~599.30, ~542.43 cm^-1^ which have been assigned to nV = O, nO-O (n1), nV-O2 (n3) and nV-O2 (n2) modes, respectively. (C) ORTEP plot with 35% probability ellipsoid of the anion of [VO(O2)2dmpz]–and dmpzH+ cation, with selected bond distances (Å): V1−O1, 1.5919 (1); V1−O2, 1.861(1); V1−O3, 1.853(1); V1−O4, 1.894(1);V1−O6, 1.1.588(1); V1−N1, 2.104(1); O1−O2, 1.480(2); O4−O3, 1.461(2); N1−N2, 1.360(2); N1−C3, 1.335(2); N2−C1,1.341(2).

Since 1899 vanadium had been used for diabetes treatment [21] as the only compound available for this purpose until the discovery of insulin [22, 23]. Several vanadium compounds have been reported over the years for their anti-diabetic activities. However, toxicity of such compounds is a concern. Some peroxyvanadium compounds were prepared [24, 25] and the compound prepared by Crans *et al*. [26] showed considerable anti-diabetic activity with minimized toxicity, but was not stable as it denatured without deep refrigeration. Hence, to retain the anti-diabetic activity, ensure stability at room temperature and render it free from toxicity became the major challenges to come up with a vanadium compound as a suitable alternative to insulin. We therefore prepared a peroxyvanadate compound i.e. DmpzH[VO(O_2_)_2_(dmpz)], henceforth referred as dmp, which is soluble in water, stable at room temperature and free from toxicity. The IR and the Raman spectra of the complex are shown in Fig 1A and 1B respectively. The IR spectra show bands at ∼944, ∼877, ∼602 and ∼530 cm^-1^ which have been assigned to ν_V = O_, ν_O-O_ (ν_1_), ν_V-O2_ (ν_3_) and ν_V-O2_ (ν_2_) modes, respectively. The spectral pattern originating from the presence of dmpz ligand is quite representative of its monodentate coordination to the metal center [27, 28]. Importantly, a positive shift of the ν_C-N_ (pyrazole ring) band by about 12 cm^-1^ compared to that of the free ligand suggests that the tertiary ring nitrogen possibly provides the binding site. This contention gains further support from the appearance of a band at *ca*. 3240 cm^-1^ owing its origin to ν_N-H_. In other words, it is the non-protonated nitrogen that appears to be bonded to the V (V) center. Like IR spectroscopy, Raman spectroscopy is also an important tool to characterize peroxo complexes of metal. The structurally significant Raman bands are given in Fig 1B. The spectra shows strong bands at ∼988,∼885.00,∼596, ∼540cm^-1^ which have been assigned to ν_V = O_, ν_O-O_ (ν_1_), ν_V-O2_ (ν_3_) and ν_V-O2_ (ν_2_) modes, respectively. From the vibrational spectroscopic data we can infer that bidentate peroxide coordination creates a local C_2v_ environment which has three IR active modes: the symmetric O-O stretch, the symmetric metal-peroxo stretch, and the asymmetric metal-peroxo stretch [29, 30] occur at *ca*. 881, 599 and 542 cm^-1^. The IR and Raman spectroscopic data compliment the X-ray structure of the compound. The molecule crystallizes in the monoclinic system P2(1)/c with four molecules in the unit cell. X-ray structure of dmp and its supporting information are provided in Fig 1C. Interestingly, this is one of the very few examples of a hexa-coordinated peroxyvanadate(V) complex. The results of X-ray experiment showed it to consist of a discrete peroxyvanadate(V) anion with one dmpz ligand, and another dmpzH^+^ as the counter cation.

### dmp binding to IR

**Fig 2:**
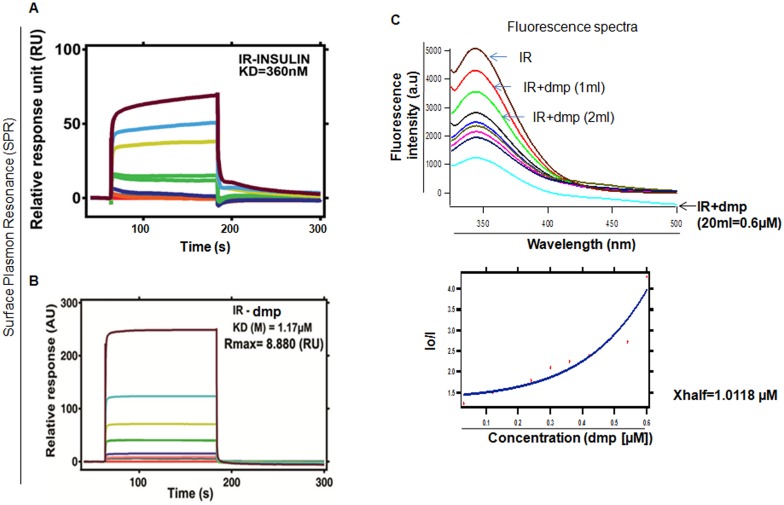
Interaction between dmp and IR. (A) Binding of recombinant insulin with insulin receptor (IR) was studied by Surface Plasmon Resonance (SPR) using varied concentrations of insulin. (B) Binding of dmp to IR was studied by SPR where increasing concentrations of dmp from 1–50 μM were flowed over immobilized IR protein on CM5 chips. Binding affinity of dmp to IR is represented by KD value (1.17 μM). Rmax value is 8.880 (RU) which represents maximum binding capacity of dmp with IR. (C) Fluorescence spectra of IR-dmp. All steady-state fluorescence measurements were carried out using an excitation wavelength of 280 nm. The emission spectra were traced from 300 to 500 nm. The concentration of IR was 0.3μM whereas varied concentrations of dmp was used for fluorescence spectra, it was gradually increased from 0–0.6μM. Binding constant was calculated from Stern–Volmer equation Io/I = 1+Ksv [Q] [dmp]. Quenching constant was Xhalf = 1.0118 μM calculated accordingly where Io and I are fluorescence intensities in the absence or presence of the quencher (dmp) respectively and Ksv was quenching constant.

Association of small insulin mimetic molecule with IR followed by its activation has been reported previously by many authors [31–35]. However, a direct binding to IR by a small molecule as its ligand is still not available. Prior to run dmp for studying its binding to IR by Surface Plasmon Resonance (SPR), we performed a standard run with insulin where IR protein was immobilized on CM5 chips. Insulin was flowed over immobilized IR from 100–1000 nM and representative sensogram obtained from there showed a KD value of 360 nM (Fig 2A). This data served as positive control. We then studied the nature of dmp and IR complex by performing SPR where increasing concentrations of dmp from 1–50μM were flowed over through immobilized IR protein on CM5 chips and resulting sensogram demonstrated equilibrium binding of dmp to IR, the KD value of this binding was found to be 1.17μM, suggesting a meaningful interaction between dmp and IR. According to the 1:1 binding model, the Rmax value is 8.880(RU) which represents an appreciable maximum binding capacity of dmp with IR (Fig 2B). SPR response correlates with change in mass concentration which depends on the molecular interaction of dmp in relation to the number of sites present in IR. SPR results were further evaluated by quantitative analysis of the interaction between IR and dmp through fluorimetric titration. 0.3μM of IR solution was titrated by successive addition of dmp to reach a final concentration of 0.6μM. Fluorescence intensity gradually quenched due to the interaction between IR and dmp. Binding constant was found to be 1.0118μM which is close to SPR value (Fig 2C).

### dmp stimulates insulin signaling pathway

**Fig 3:**
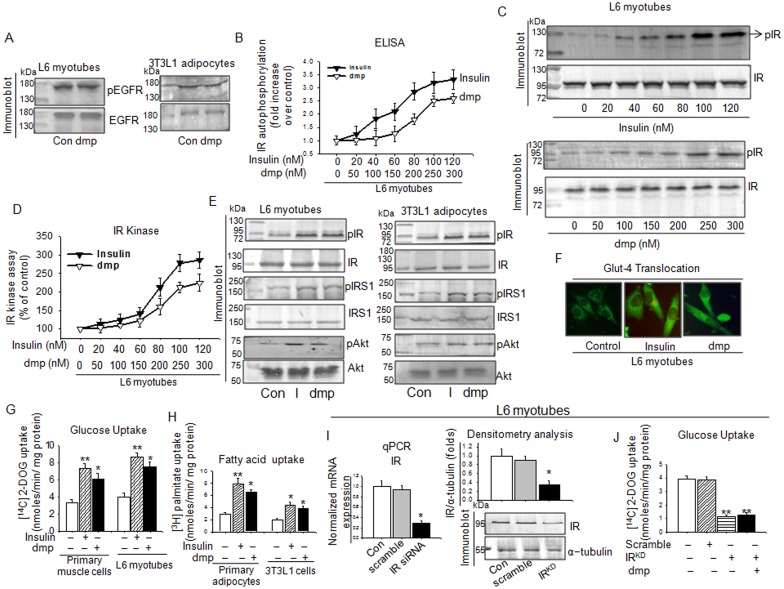
dmp binding to IR augments insulin signalling pathway. (A) dmp fails to activate EGFR. L6 myotubes or 3T3L1 adipocytes were treated with or without 250 nM dmp for 4h. The cell lysates were analyzed by immunoblotting with anti-pEGFR and anti-EGFR antibodies. (B,C) dmp can induce IR phosphorylation in a dose dependent manner. L6 myotubes were treated with insulin (20–120 nm) or dmp (50–300 nm) for 4h and IR phosphorylation was monitored by ELISA (B) or immunoblotting with anti-pIR and anti-IR antibodies (C). (D) IR kinase activity was determined in L6 myotubes which were incubated with varied concentrations of insulin or dmp. (E) dmp stimulates IR and its downstream kinases phosphorylation. L6 myotubes or 3T3L1 adipocytes were treated with or without Insulin (100 nM) or dmp (250 nM) for 4h and the IR phosphorylation and its downstream signalling were monitored by immunoblotting. (F) L6 myotubes transfected with GFP-GLUT4 chimeric gene were incubated with insulin (100 nM) or dmp (250nM) for 4h. Cells on the cover slips were fixed in paraformaldehyde and observed under florescent microscope for GFP-GLUT4 translocation. (G) dmp like insulin promotes glucose uptake. L6 myotubes or skeletal muscle cells from soleus muscle of neonatal mice (2-3days) were incubated with 100 nm insulin or 250 nm dmp for 25 min. [^14^C] 2-DOG was then added, and the cells were further incubated for 5 min. [^14^C] 2-DOG uptake was measured by scintillation counting. *P<0.05 *versus* Con; **P<0.01 *versus* Con. (H) dmp augments fatty acid uptake. Primary culture adipocytes or 3T3L1 adipocytes were treated with 100 nm insulin or 250 nm dmp for 4h followed by incubation with [^3^H] Palmitate for 15 min. [^3^H] Palmitate uptake was measured in a liquid scintillation counter. *P<0.05 *versus* Con; **P<0.01 *versus* Con. (I) L6 myotubes were tranfected with IR siRNA(IR^KD^) followed by estimation of IR gene and protein levels by qPCR (left) and immunoblotting (right) respectively. *P<0.05 *versus* Con. (J) IR^KD^ L6 myotubes were incubated with dmp for 4h and [^14^C] 2-DOG uptake was measured according to the above description. **P<0.01 *versus* Con. All values are represented as mean ± s.e.m. (n = 5).

The specificity of dmp binding to IR was examined by observing its binding to EGFR. Fig 3A demonstrates that dmp did not recognize EGFR. Autophosphorylation of IR in L6 myotubes could be dose dependently increased by both dmp and insulin. 100 nM insulin produced highest effect while it was 250 nM in the case of dmp (Fig 3B and 3C). Interestingly, both dmp and insulin were found to be active at nM dose, this was not observed with other insulin mimetic compounds which were active at μM dose in *in vitro* experiments [31–35]. Similar trend was observed with tyrosine kinase activity of IR by dmp and insulin (Fig 3D). A comparison between insulin and dmp was then examined with insulin downstream molecules, both stimulated the downstream kinases (Fig 3E) to effect translocation of GLUT4 from cytosol to membrane. Fig 3F represents that in GFP-GLUT4 transfected myotubes insulin and dmp induced GLUT4 translocation. These results indicate that dmp mimics insulin effects starting from IR activation to GLUT4 translocation. The same was also reflected in glucose uptake by primary muscle cells and L6 myotubes (Fig 3G). Both insulin and dmp significantly stimulated fatty acid uptake by adipocytes (Fig 3H). L6 myotubes were transfected with IR siRNA and to assess the amount of IR gene suppression due to this silencing we determined the IR gene and protein levels which show a significant suppression with 71% in gene and 63% in protein as compared to respective controls (Fig 3I). IR activation by dmp that led to uptake of glucose was significantly attenuated due to knockdown of IR gene in L6 myotubes (Fig 3J) indicating that dmp’s stimulatory effect was mediated through IR.

### dmp mimics insulin effects in insulin deficient mice

**Fig 4:**
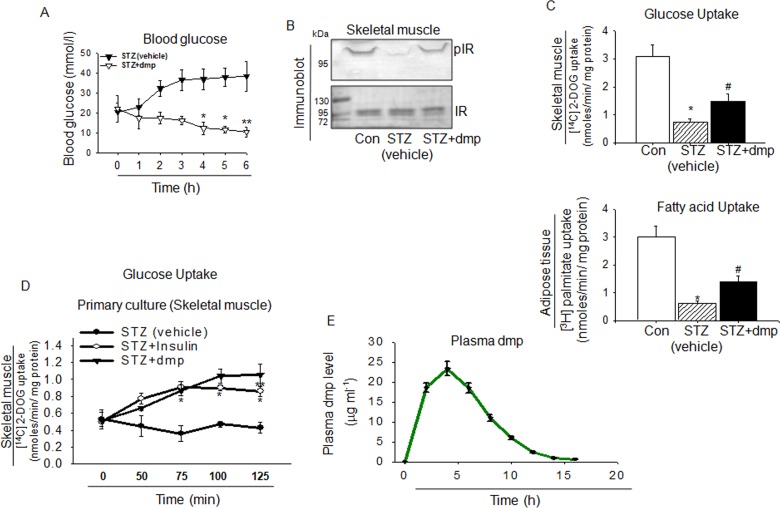
In streptozotocin induced diabetic mice dmp acts like insulin. dmp activates insulin signaling in STZ mice. STZ induced BALB/c mice were starved for 12 h followed by oral administration of dmp (300μg kg^-1^ bw) or vehicle. (A) Blood glucose level was detected at different times. *p< 0.05 *versus* STZ (vehicle), **p< 0.01 *versus* STZ (vehicle). (B) 4 h after dmp treatment, skeletal muscle tissues were collected from BALB/c mice (Con) or STZ mice or STZ mice fed with dmp, lysed and subjected to immunoblot using anti-pIR and anti-IR antibodies. (C) [^14^C] 2-DOG uptake (top) and [^3^H] Fatty acid uptake (bottom) by skeletal muscle or adipose tissue from above mentioned mice were determined in a liquid scintillation counter. *P<0.05 *versus* Con; #*P*<0.05 *versus* STZ (vehicle). (D) Skeletal muscle from STZ mice was incubated with insulin (100 nM) or dmp (250 nM) and [^14^C] 2-DOG uptake was measured at different time intervals. *P<0.05 or **P<0.01 *versus* STZ (vehicle). (E) dmp was orally administered to BL6 mice (5mg kg^-1^ bw) and plasma dmp level was measured at different time intervals. All values are represented as mean ± s.e.m. (n = 5).

Streptozotocin induced destruction of β-cells in mice cause drastic fall in endogenous insulin that consequently increased blood glucose level. Fig 4A shows that in STZ induced Type1 diabetes mice blood glucose level was markedly elevated while oral administration of dmp could reduce it significantly. Decrease of skeletal muscle IR phosphorylation was also improved by dmp (Fig 4B). Same was observed with glucose uptake by skeletal muscle and fatty acid uptake by adipose tissue of STZ treated mice, dmp treatment considerably improved the defects caused due to the dearth of insulin (Fig 4C). The dmp dose that we used for *in vivo* treatments was 300μg kg bw^-1^ whereas it was at mg/kg level by other insulin mimetic compounds [31–35]. Another advantage of dmp was that its stimulatory effect on [^14^C] 2-DOG uptake could be noted for longer period whereas with insulin the plateau was reached at 75 min (Fig 4D). Pharmacodyanamics study of dmp demonstrated that its presence in the blood after oral administration (5mg kg bw^-1^) could be detected at 1 h, peak reached at 5 h and it was retained till 7 h (Fig 4E). Maximum concentration (Cmax) in plasma was 23.3 μg ml^-1^. These findings suggest that bioavailability of dmp would not be a problem.

### dmp regulates PPAR_ϒ_ and its target gene expression

**Fig 5:**
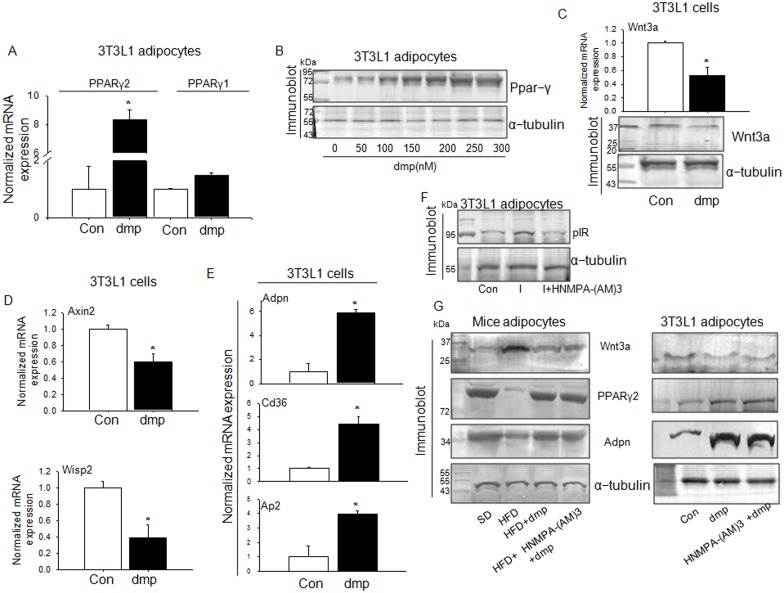
dmp induces expression of Ppar-γ and its target genes through the suppression of Wnt3a. *In vitro* incubation of 3T3L1 cells were conducted in the absence (control) or presence of 250 nM dmp along with 0.5mM palmitate for 4 h. (A) RNA was extracted from cells and Ppar-γ1 and Ppar-γ2 mRNA level was measured by quantitative PCR. *P<0.001 *versus* Con (B) dmp effects a dose dependent increase in PPARϒ expression. The cell lysates were used for immunoblotting with anti-PPARϒ antibody or anti-α tubulin antibody for loading control. (C) Wnt3a gene (top) and protein (bottom) expression levels were estimated. *P<0.01 *versus* Con. (D) mRNA expressions of Wnt target genes, axin2 and wisp2 were estimated. *P<0.01 *versus* Con. (E) dmp effect on mRNA expression of PPARϒ target genes Adpn, CD36 and aP2 in 3T3L1 adipocytes was observed. *P<0.01 *versus* Con. (F) 3T3L1 adipocytes were incubated with 100 nM insulin (I) in the absence or presence of insulin receptor tyrosine kinase inhibitor HNMPA-(AM)3 and pIR protein levels are estimated by immunobloting. (G) Wnt3a, PPARϒ2 and Adpn protein levels were estimated in primary mice adipocytes from SD and HFD mice treated with or without dmp (250 nM) or dmp+HNMPA-(AM)3 (100 μM) (left). 3T3L1 adipocytes were preincubated with HNMPA-(AM)3 for 1 h followed by addition of 0.5mM palmitate in the absence or presence of dmp for 4 h to determine Wnt3a, PPARϒ2 and Adpn protein levels through immunoblot analysis. All values are represented as mean ± s.e.m. (n = 5).

An interesting dimension of insulin activity is its regulatory role on PPARγ expression. Insulin deficiency effects a decrease in PPARγ gene expression in the adipose tissue of rodents which could be reversed by insulin treatment [36]. Abnormalities in PPARγ expression has been found to be associated with the loss of insulin sensitivity in obesity and Type2 diabetes [37,38] indicating insulin regulation of PPARϒ expression. Direct evidences have shown that insulin could induce PPARϒ gene and protein expression in human adipocytes [39]. This excess expression of PPARγ has been reported to be associated with increase in insulin sensitivity [40], a function known to be performed by PPARγ ligands. We therefore hypothesized that dmp may also enhance PPARγ expression as it mimics insulin activity. To test this, we performed experiments with 3T3L1 adipocytes. Fig 5A shows that dmp could significantly induce PPARγ gene expression in 3T3L1 adipocyte, it preferably expressed PPARγ2 while PPARγ1 was only marginally increased over the control (Fig 5A). Determination of PPARγ protein expression with varied doses of dmp also exhibited a dose dependent increase of PPARϒ protein (Fig 5B). In addressing the question about how dmp could augment PPARϒ expression, the first possibility seems to be the suppression of Wnt signaling. Emerging evidences from several laboratories indicated that PPARγ is regulated by Wnt3a [40–42]. Fig 5C demonstrates that dmp significantly reduced Wnt3a gene and protein expression. It also decreased the expression of Wnt target genes i.e. axin2 and wisp2 (Fig 5D), and that permitted PPARϒ expression which in turn enhanced adiponectin, Cd36 and aP2 expression (Fig 5E). Since dmp is an insulinomimetic compound and insulin also augments PPARϒ expression [39], this increase in PPARϒ may be attributed as its insulin like effect and not related to insulin sensitization. The meaningful way to demonstrate that dmp’s effect is independent of insulin is to block IR and then observe whether dmp could enhance PPARϒ expression. We checked dmp induced suppression of Wnt3a because this is responsible for augmenting PPARϒ level. A peptide inhibitor of IR which efficiently blocks insulin induced IR activation i.e. HNMPA-(AM)3 [11, 43] was used by us to show dmp’s IR independent effect. We examined this inhibitor in 3T3L1 incubation where HNMPA-(AM)3 strongly inhibited insulin induced IR activation (Fig 5F). We therefore used HNMPA-(AM)3 to block IR in different experiments with dmp. Fig 5G demonstrates the results obtained with primary mice adipocytes, there was a significant increase of Wnt3a expression in HFD mice adipocytes as compared to SD mice adipocytes, dmp markedly suppressed it and this effect remained undisturbed when IR was blocked with a peptide inhibitor, HNMPA-(AM)3, indicating that dmp inhibition of Wnt3a is not mediated through IR. Similar results were obtained with PPARϒ and adiponectin where dmp’s stimulatory effect on them was not altered due to IR inhibition (Fig 5G). This was expected because suppression of Wnt3a permits PPARϒ and adiponectin expression. dmp’s insulin sensitizing effect through the elevation of PPARϒ and adiponectin was further investigated with 3T3L1 adipocytes. Here also suppression of Wnt3a by dmp and corresponding increase in PPARϒ and adiponectin levels were found to be not mediated through IR as inhibition of IR by HNMPA-(AM)3 did not interfere with dmp’s effects (Fig 5G).

### dmp induced adiponectin elevation in db/db mice promotes insulin sensitivity

**Fig 6:**
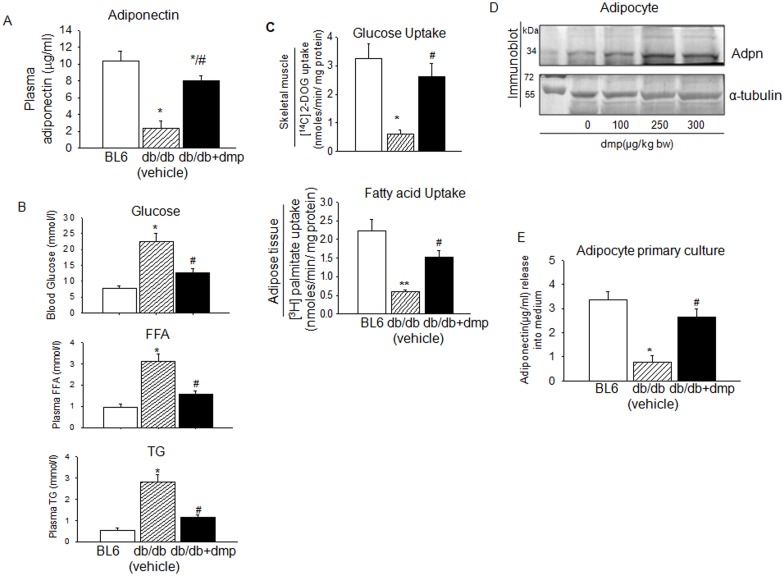
dmp stimulation of adiponectin causes improvement of insulin sensitivity. (A) *db/db* mice were orally administrated with dmp (300 μg kg^-1^ bw) or vehicle for 28 days. Adiponectin level in plasma of BL6, *db/db* (vechile) and dmp fed *db/db* mice was estimated by ELISA. *P< 0.05 versus BL6; #*P*<0.05 *versus db/db* (vechile) mice. (B) BL6, *db/db* (vechile) and dmp fed *db/db* mice were starved for 12h and blood glucose, plasma FFA and triglyceride (TG) levels were estimated. *P< 0.05 versus BL6; #*P*< 0.05 *versus db/db* (vechile). (C) [^14^C] 2-DOG uptake (top) and [^3^H] Palmitate (bottom) uptake by skeletal muscle and adipose tissue of BL6, *db/db* (vechile) and dmp fed *db/db* mice were measured in a liquid scintillation counter. *P<0.05 *versus* BL6; #*P*<0.05 *versus db/db* (vechile). (D) *db/db* mice were orally administrated with vehicle or dmp (100–300 μg kg^-1^ bw) for 28 days. Adipocytes were collected, lysed and immunobloted with anti-adpn and anti-α tubulin antibodies to detect protein expression. (E) 250nM dmp was added to primary culture of adipocytes isolated from *db/db* mice, incubated for 4h and release of adiponectin into the culture medium was estimated by ELISA. *P<0.05 *versus* BL6; #*P*<0.001 *versus db/db*. All values are represented as mean ± s.e.m (n = 5).

**Fig 7:**
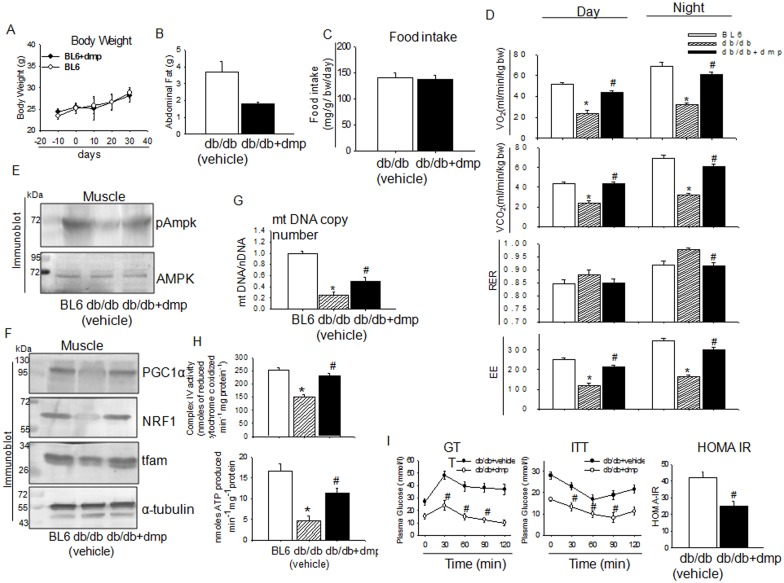
dmp improves energy homeostasis in *db/db* mice. (A) BL6 mice were orally administrated with vehicle or dmp (300 μg kg^-1^ bw) for 28 days. Body weight was recorded on the days mentioned in the figure. (B) *db/db* mice were orally administered with vehicle or dmp (300 μg kg^-1^ bw) for 28 days. Weight of the abdominal fat was recorded. (C) Food intake was estimated in *db/db* (vechile) and dmp fed *db/db* mice. (D) Metabolic activities were measured by indirect colorimetry in BL6, *db/db* (vechile) and dmp fed *db/db* mice during day and night periods. The experimental animals were placed in metabolic cage and average hourly oxygen consumption (V̇O_2_) and carbon dioxide production (VCO_2_) were measured. Accordingly, RER and energy expenditure (EE) were calculated. *P<0.05 *versus* BL6; #*P*<0.05 *versus db/db* (vechile). (E) Western blot showing phosphorylation status of pAMPK and AMPK in the skeletal muscle of BL6, *db/db* (vechile) and dmp fed *db/db* mice. (F) Immunoblots showing abundance of PGC1α, NRF1 and tfam level in dmp treated mice. (G) Total DNA was extracted from muscle tissue and the content of mtDNA was calculated using real-time quantitative PCR by measuring the threshold cycle ratio (㥆Ct) of a mitochondrial-encoded gene COXII versus a nuclear encoded gene RIP140. *P<0.05 *versus* BL6; #*P*<0.05 *versus db/db* (vechile). (H) Complex IV activity (top) and ATP production (bottom) was measured in mitochondria isolated from skeletal muscle of BL6, *db/db* (vehicle) and dmp fed *db/db* mice. *P<0.05 *versus* BL6; #*P*<0.05 *versus db/db* (vechile). (I) *db/db* mice were orally administrated with vehicle or dmp (300 μg kg bw^-1^) for 28 days. Blood glucose concentration (GTT) was measured before and after oral gavages of 1g glucose kg bw^-1^ at the indicated time points. ITT was performed after injecting mice with 0.7U insulin kg bw^-1^. Fasting insulin and fasting glucose was estimated and HOMA-IR was calculated. #*P*<0.05 *versus db/db* (vechile). All values are represented as mean ± s.e.m. (n = 5).

Results described above have shown that dmp could induce adiponectin expression through enhanced expression of PPARϒ. The same was also reported with insulin [39, 44]. There was depleted adiponectin level in the plasma of *db/db* mice which was reversed by the administration of dmp (Fig 6A). This coincided with the lowering of blood glucose, free fatty acid (FFA) and TG levels (Fig 6B) and increased the uptake of glucose and fatty acid by skeletal muscle and adipose tissue respectively (Fig 6C). Orally fed dmp also increased adiponectin expression in the adipose tissue in a dose dependent manner (Fig 6D). dmp addition to the primary culture of adipocyte from *db/db* mice significantly increased adiponectin release in the medium (Fig 6E).

dmp administration did not alter body weight in BL6 mice (Fig 7A) and considerably reduced abdominal fat mass in *db/db* mice (Fig 7B) which indicates increase in energy expenditure because there was no difference in calorie intake (Fig 7C). Oral administration of dmp significantly improved O_2_ consumption (VO_2_) and carbon dioxide production (VCO_2_) during the day and night periods (Fig 7D). Mice being a nocturnal animal, during the night time when they are more active, respiratory exchange ratio (RER) was found to be lower in dmp fed *db/db* mice suggesting that a higher amount of fat was utilized by dmp treated mice for their energy production than untreated *db/db* mice (Fig 7D). Administration of dmp significantly improved energy expenditure (EE) (Fig 7D) indicating that dmp could prevent diet induced impairment of energy homeostasis. Adiponectin is known to regulate AMPK activation which is the master regulator of energy homeostasis [45]. Skeletal muscle tissue from dmp treated *db/db* mice showed a significant increase in AMPK activation (Fig 7E) which markedly augmented mitochondrial biogenesis, bioenergetics and ATP synthesis (Fig 7F, 7G and 7H) indicating a greater utilization of stored energy because of dmp. This together significantly improved insulin sensitivity as would be evident from GTT, ITT and HOMA-IR (Fig 7I).

### Toxicity study of dmp

**Fig 8:**
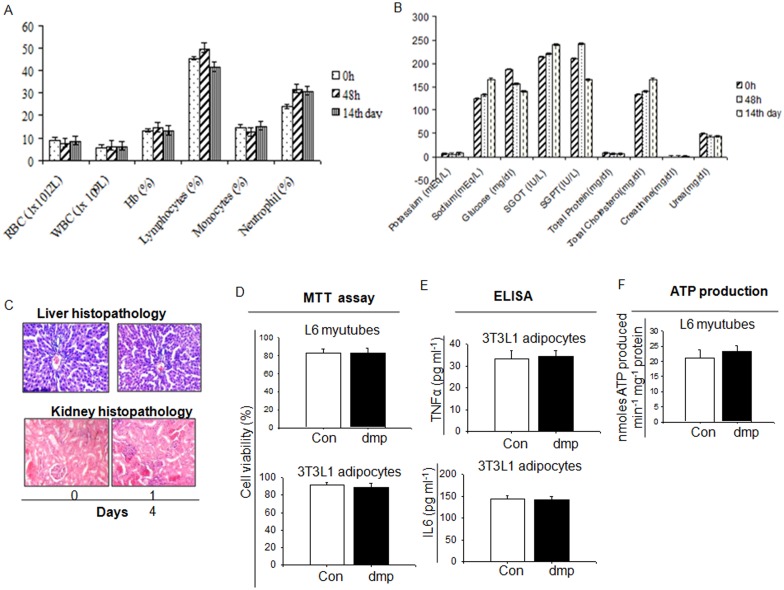
Toxicity test of dmp. Rats were fed with dmp (5 mg kg bw^-1^) for 14 days. (A) Hematological, (B) Biochemical analysis, (C) Liver and kidney histopathology were performed on 0 and 14 day to test toxic effect of dmp. (D) L6 myotubes and 3T3L1 adipocytes were incubated with 300 nM of dmp for 4 h and cell viability was assessed by MTT assay. (E) After incubation of 3T3L1 adipocytes with or without 300 nM dmp for 4 h, release of TNFα and IL6 in to the medium was estimated through ELISA. (F) ATP production was measured in L6 myotubes incubated in the absence or presence of 300nM dmp for 4 h. All values are represented as mean ± s.e.m. (n = 5)

Toxicity of dmp (single dose-5mg kg bw^-1^) to Wistar rats (*Rattus norvegicus*) was evaluated as per the guideline of OECD-420 [46]. dmp administered rat did not show any abnormal behavior including food taking, gross body weight etc. To determine dmp toxicity blood parameters such as WBC counts, total leukocyte count, hemoglobin, total lymphocytes, monocytes and neutrophils were measured after 14th day of the treatment and it was found that all these parameters are in normal range (Fig 8A), indicating that dmp does not have any adverse effects. In addition to these parameters we have also determined some important biochemical markers of liver, kidney including lipid profile. Fig 8B demonstrates that there was no alteration of these parameters due to dmp treatment (Fig 8B). Histopathology of the liver and kidney did not show abnormality (Fig 8C). Besides *in vivo* validation of dmp toxicity, we also tested its toxic effect at cellular level. We have taken skeletal muscle cell line (L6 myotubes) and 3T3L1 adipocytes and observed whether there was any toxic effect of dmp through MTT cell viability assay, level of proinflammatory cytokines i.e. TNFα and IL6 and cellular ATP profile (Fig 8D–8F). It would be evident from the results that dmp did not show any abnormality in cell viability, there was also no notable changes in proinflammatory cytokine levels and ATP production between control and dmp treated cells (Fig 8D–8F). These findings suggest that dmp did not produce toxic effects in the above mentioned experimental models. In addition, according to the GHS (Global Harmonization system), dmp was found to be under category 2.

## Discussion

Anti-diabetic effect of vanadium salt, Na_2_VO_4_ (sodium orthovanadate) has been reported 22 years before the discovery of insulin. It is Lyonnet *et al*., [21] who reported insulin-like activity of vanadium in 1899 when administered orally. Several studies followed thereafter showed various insulin-mimetic effects of vanadium compounds both *in vitro* and *in vivo*, which include stimulation of glucose transport and glucose oxidation [47, 48] glycogen synthesis and lipogenesis [49]. In animal experiments, glucose homeostasis in diabetic rats [50], normalization of hyperglycemia in diabetic mice [51] and insulin sensitization effect in Type2 diabetes patients [52] are few examples from numerous reports on anti-diabetic effects of various vanadium compounds. Primary reason for which vanadium compounds are not recommended for clinical therapy is their toxic side effects. The toxic side effects of Vanadium are reported by several authors which includes dehydration, hepatic and renal toxicity [53]. Some Vanadium compounds have been found to interact with DNA and have considerable toxicity [54]. Domingo *et al*. [55] observed that vanadium administration to rats decreased weight gain, and increased serum concentrations of urea and creatinine. To use vanadium as a therapy because it showed significant efficacy in dealing with diabetic problems, attempts were made by several investigators to minimize the toxicity by preparing a peroxy vanadium compound which showed encouraging results. One of such compounds has been prepared by Crans *et al* [26], that showed considerable increase in anti-diabetic activity with minimized toxicity, but its stability remains a problem. Major issues those are concerned with the use of vanadium salt for diabetes treatment is to fulfill the following requirements: (a) it should possess high sensitivity so that even a low dose can produce desired anti-diabetic effects, (b) free from toxicity, (c) stable at room temperature, (d) better retention in the circulation and (e) reasonable bioavailability when administered through oral route. To meet these objectives, we prepared a hexacordinated peroxyvanadium(V) compound with 3,5-dimethyl imidazole(dmp) ligand. Dmp could satisfy these requirements with significantly high insulin mimetic activity and improve the loss of insulin sensitivity that occurs in Type2 diabetes. dmp is a novel vanadium compound, distinctly varies from other vanadium compounds examined so far for insulin like activities. By binding to IR insulin produces both metabolic as well as mitogenic effects, while dmp only showed metabolic activities, it does not exhibit mitogenic activity as evident from unaltered EGFR expression and activation. dmp binding to IR causes IR activation which in turn triggers downstream signaling cascade including translocation of GLUT4 to the skeletal muscle cell membrane. Previous reports with vanadium described translocation of GLUT4 for increasing glucose transport, without altering GLUT4 transcription or protein expression [56]. We also could not detect increase in GLUT4 expression with dmp (data not shown). It possibly effects efficient translocation of GLUT4 due to the increase in intrinsic activity.

Vanadium inhibitory effect on non-specific protein tyrosine phosphatase (PTPase) has been focused as one of its major function which permitted its insulin like activity [57]. This indicates that vanadium effect on IR activation has been indirectly mediated [57]. Although vanadium inhibitory effect on PTPase is important in mediating the metabolic effects, number of evidences suggests that inhibition of PTPase is insufficient to cover plethora of activities by various vanadium compounds. Vanadium effects glycogen metabolism and gluconeogenesis, lipogenesis, insulin sensitization in Type 2 diabetes etc. and some of these activities do not involve PTPase [53, 58, 59]. If vanadium compound’s insulin mimetic effect is only because of its inhibition of PTPase 1B, then it would always depend on insulin induced activation of IR. But some studies with vanadium demonstrate its stimulatory effect could be independent of insulin or IR activation [47, 58, 59]. Again, such multifunctional actions of vanadium compounds have shown preferential increase in metabolic effects [60]. It is indeed difficult to consider the view that such various kinds of activities executed by different vanadium compounds belong to post receptor effects. Demonstration of dmp binding to IR in this report is a strong evidence in favour of this. Moreover our contribution will provide a better understanding of vanadium compound-induced insulin mimetic action, a search for which is continuing for last 45 years.

We have demonstrated that dmp significantly enhanced insulin sensitivity in obese diabetic mice. Previous reports showed that vanadyl sulphate could markedly improve insulin sensitivity of muscle and liver in Type 2 diabetes human patients [52]. Insulin sensitizing effect of vanadium has also been examined in animal models, administration of it has been found to increase insulin sensitivity in diabetic mice and rat models [51,56]. However, underlying mechanism involved in insulin sensitization by vanadium compounds remains unclear. We have demonstrated that dmp increases PPARϒ expression in the adipocyte through the inhibition of Wnt signaling. Wnt3a has been shown to suppress PPARϒ and its target gene expression [40], for this reason it inhibits adipocyte differentiation [41]. dmp efficiently overcomes this obstacle by inhibiting Wnt 3a along with its target genes axin2 and wisp2 and that permitted PPARϒ expression. This excess PPARϒ enhances the expression of adiponectin, CD36, aP2 and that improves insulin sensitivity. Orally administered dmp regulated adverse effects due to aberrant lipid metabolism in *db/db* mice because of considerable increase in adiponectin production through PPARγ. It is intriguing to note that effects of dmp on adipocyte gene expression are similar to harmine which also regulate PPARγ and its target gene expression. It follows similar molecular action in enhancing PPARγ expression i.e. through the inhibition of Wnt signaling. Harmine is a small anti-diabetic molecule, description of its action revealed that excess of PPARγ expression in adipocyte could produce the effects similar to its ligands i.e. TZDs which attenuate lipid induced defects by controlling fat metabolism thus causing improvement of insulin sensitivity. Hence, regulators of PPARγ expression would also serve its ligand like activities but without producing adverse toxic effects witnessed with TZDs [40]. dmp, like harmine, is another small molecule that regulate PPARϒ through similar mechanism. dmp has shown a new and relevant dimension in relation to energy homeostasis. Insulin resistance in human skeletal muscle has been shown to be associated with decreased mitochondrial oxidative capacity and downregulation of genes which regulate mitochondrial activity thus resulting in decreased ATP synthesis. Number of recent reports emphasize a strong link between impaired mitochondrial function and Type 2 diabetes [61]. There is practically no compound to reverse this except resveratrol which by improving mitochondrial function could protect mice from obesity and insulin resistance [62]. In this connection dmp showed a promise, it stimulated mitochondrial biogenesis and bioenergetics through increased adiponectin and could effectively regulate energy homeostasis which has an impact on insulin sensitivity. Another important aspect of dmp is its activation of insulin signaling molecules through its binding to IR which is expected to mitigate the problems associated with β-cell destruction that occurs both in Type1 diabetes and Type2 diabetes.

dmp provides several favorable profiles to deal with Type2 diabetes and Type1 diabetes, its dose to produce insulin like effects is significantly lower as compared to other orally effective insulin mimetic molecules reported previously. In both *in vitro* and *in vivo* studies dmp produces insulin like activity in insulin target tissues or for Type2 diabetes mice with considerable low dose. Dose response study for *in vitro* and *in vivo* effects is very important because some of the insulin mimetic effects of vanadium observed in vitro failed to produce results with therapeutic doses when examined in *in vivo* experiments therefore lacks relevance for clinical application [53]. dmp has excellent solubility in water and within a short time of oral administration it could be detected in the blood and retained for 7 h, its pharmacodynamics is impressive, C_max_ is high, suggesting better bioavailability, and more importantly it does not produce toxicity as observed with animal test.
